# MMSET: Role and Therapeutic Opportunities in Multiple Myeloma

**DOI:** 10.1155/2014/636514

**Published:** 2014-07-01

**Authors:** Zhigang Xie, Wee Joo Chng

**Affiliations:** ^1^Cancer Science Institute of Singapore, National University of Singapore, 14 Medical Drive, Singapore 117599; ^2^Department of Medicine, Yong Loo Lin School of Medicine, National University of Singapore, 1E Kent Ridge Road, Singapore 119228; ^3^Department of Haematology-Oncology, National University Cancer Institute of Singapore, National University Health System, Singapore 119228

## Abstract

Recurrent chromosomal translocations are central to the pathogenesis, diagnosis, and prognosis of hematologic malignancies. The translocation t(4; 14)(p16; q32) is one of the most common translocations in multiple myeloma (MM) and is associated with very poor prognosis. The t(4; 14) translocation leads to the simultaneous overexpression of two genes, *FGFR3* (fibroblast growth factor receptor 3) and *MMSET* (multiple myeloma SET domain), both of which have potential oncogenic activity. However, approximately 30% of t(4; 14) MM patients do not express FGFR3 and have poor prognosis irrespective of FGFR3 expression, whereas MMSET overexpression is universal in t(4; 14) cases. In this review, we provide an overview of recent findings regarding the oncogenic roles of MMSET in MM and its functions on histone methylation. We also highlight some of MMSET partners and its downstream signalling pathways and discuss the potential therapeutics targeting MMSET.

## 1. Introduction

Compelling data has emerged that epigenetic changes underlie a wide variety of pathologies, including cancer [1, 2]. Epigenetic regulation includes DNA methylation and covalent histone modifications. These processes may play an important role in the initiation and progression of many cancers, including the haematological malignancy multiple myeloma (MM).

**Figure 1:**
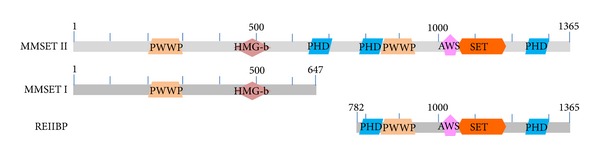
*Schematic primary structure of three major MMSET isoforms*. PWWP, named for a conserved Pro-Trp-Trp-Pro motif; HMG-b, high mobility group (HMG) box domain; PHD, plant homeodomain zinc finger; AWS, associated with SET domain; and SET, lysine methyltransferase catalytic domain. Conserved domain architecture was analyzed through http://blast.ncbi.nlm.nih.gov.

MMSET (multiple myeloma SET domain), also known as Wolf-Hirschhorn syndrome candidate 1 (WHSC1) or nuclear receptor-binding SET domain 2 (NSD2), is a member of the NSD histone methyltransferase (HMT) family also including NSD1 and NSD3 [3–5]. The* MMSET* gene spans 120 kb, consists of 24 exons, and undergoes complex alternative splicing. Two major transcripts were identified: type I encodes a protein of 647 amino acids and type II encodes a protein of 1365 amino acids. Both proteins share a common amino terminus [6]. A third transcript initiated within a middle intron of* MMSET* encodes a protein named RE-IIBP [7] (Figure 1). Conserved domain architecture analysis indicated that MMSET is a multidomain protein containing a set domain (determining protein lysine methyltransferase activity), 2 PWWP (named for a conserved Pro-Trp-Trp-Pro motif) domains, a HMG (high mobility group) box, and 3 PHD (plant homeodomain) fingers (Figure 1). The PWWP, HMG, and PHD domains are responsible for nuclear localization, DNA-binding, and recognition of histone marks [8–11]. The importance of MMSET in malignancy was first highlighted by characterization of the t(4; 14) translocation in about 15% of MM, which fuses the* MMSET* gene to the immunoglobulin heavy-chain promoter/enhancer, leading to dramatic upregulation of MMSET expression [6, 12].

## 2. Oncogenic Role of MMSET in MM

Recurrent chromosomal translocations are central to the pathogenesis, diagnosis, and prognosis of hematologic malignancies. In the past decade, it has become apparent that approximately 50% of MM harbor recurrent translocations involving the immunoglobulin heavy chain (IgH) locus on chromosome 14q32 [13–15]. The translocation t(4; 14)(p16; q32) is one of the most common translocations in MM, affecting 15% of patients, and is associated with very poor prognosis [16]. The t(4; 14) translocation leads to the simultaneous overexpression of two genes,* FGFR3* (fibroblast growth factor receptor 3) and* MMSET*. FGFR3 has transforming activity* in vitro* and* in vivo*, but approximately 30% of t(4; 14) MM patients do not express FGFR3, whereas overexpression of MMSET isoforms is a universal feature of t(4; 14) cases [12, 17, 18]. Furthermore, the poor prognosis of t(4; 14) persists irrespective of FGFR3 expression [12]. These data suggest that MMSET may be the critical oncogene in this translocation. Downregulation of MMSET expression in MM cell lines indicated that t(4; 14) MM cells rely on MMSET expression for clonogenic growth and tumorigenicity* in vivo *[13]. These results provide the first direct evidence that MMSET is an oncogene and plays a significant role in t(4; 14) MM. Later several groups reported that depletion of MMSET could also inhibit proliferation and induce cell cycle arrest and apoptosis [19–22]. Consistent with MMSET knockdown results, wild type MMSET, but not the MMSET catalytic mutants, could restore proliferation and colony formation of t(4; 14) MM cells upon MMSET deletion. Furthermore, complementation of MMSET knockout cells with wild type MMSET instead of catalytic mutants could restore their tumorigenicity* in vivo *[10, 22]. These data further conformed that MMSET is an oncogene and its oncogenic role is dependent on its catalytic activity. Recent study has shown that MMSET mRNA level is also upregulated in 15 of 40 tumor types compared to their normal tissue counterparts. Furthermore, MMSET mRNA levels are associated with tumor aggressiveness or prognosis in several of these tumors [23]. Thus, in addition to t(4; 14) MM, MMSET may contribute to the development of other cancer types.

## 3. MMSET Function on Histone Methylation

Histones are the stage of diverse posttranslational modifications that ultimately regulates the gene transcription. Lysine methylation is one prominent feature of the posttranslational histone modifications in the regulation of chromatin structure and function. Lysine-HMTs target specific histone residues on H3 and H4 and can transfer one, two, or three methyl groups on specific lysines on the histone tails [24]. The MMSET protein contains AWS (associated with set) SET-PostSET domains that are highly conserved with yeast H3K36-specific methyltransferase Set2 [25]. It is first reported that the MMSET protein is a H4K20 methyltransferase with characteristics of a transcriptional corepressor [19]. Later, several reports suggested that MMSET could generate numerous different histone marks, including H3K4me2, H3K9me2, H3K27me3, H3K36me2, H3K36me3, and H4K20me2 [26–30]. Biologically, MMSET is reported to repress transcription through generation of H4K20me3 [19], H3K27me3 [27], or H3K36me3 [29], to enhance transcription through generation of H3K4me2 [26] and H3K36me2 [21], and to mediate accumulation of 53BP1 to DNA damage sites through generation of H4K20me2 [30]. Recently multiple independent biochemical and cellular approaches were used to investigate and resolve the discrepancies regarding MMSET enzymatic activity. It is suggested that the principal physiologic activity of MMSET at chromatin is dimethylation of H3K36, and in the process rules out generation of H3K36me3, H4K20me2, and several other putative methyl products of MMSET [22]. This conclusion is in agreement with a study reporting* in vitro* dimethylation activity at H3K36 by the three NSD family members NSD1, NSD2, and NSD3/WHSC1L1 [28].

## 4. MMSET Interacting Proteins and Downstream Targets

A preliminary step in understanding protein structure and function is to determine which proteins interact with each other, thereby identifying the relevant biological pathways. MMSET is a multidomain protein containing a catalytic set domain and other highly conserved domains, such as PWWP, PHD, and HMG-box, to mediate chromatin interaction and recognition of histone marks. Coprecipitation experiments indicated that MMSET could interact with histone modifiers HDAC1, HDAC2, and LSD1 [19, 31]. SET domain containing methyltransferases seems to be particularly sensitive to the sequence and posttranslational modifications surrounding the target lysine site [32]. Hence, the histone modification signature of MMSET is complex and might be mediated both by direct action of MMSET and by interplay with other histone modifiers. To determine the genes regulated by MMSET, Martinez-Garcia et al. profiled gene expression in the loss-of-function (MMSET knockdown) and gain-of-function (MMSET reexpression in the MMSET knockout cell line) systems [21]. Signaling pathway analysis indicated that MMSET could regulate cell death and the p53 pathway (e.g., BAX, BCL2, and caspase 6), the cell cycle (cyclin E2, E2F2, TP53INP1, and CDC25A), genes for DNA repair (ATM, E2F2, and GADD45A), and integrin-mediated signaling (CDC42 and integrin alpha-L). These results are consistent with phenotypes of proliferation inhibition and apoptosis induction upon MMSET knockdown in t(4; 14) MM cells. Recently a microRNA (miRNA) profiling in t(4; 14) MM cells identified miR-126* as an MMSET-regulated miRNA, which could specifically target the 3′-untranslated region of c-Myc and inhibit its translation [33]. Moreover, the expression of miR-126* was sufficient to decrease the proliferation rate of t(4; 14) MM cells. Chromatin immunoprecipitation (ChIP) analysis showed that MMSET binds to the miR-126* promoter along with the KAP1 corepressor and histone deacetylases to repress miR-126* transcription. Through quantitative mass spectrometry analysis we found that overexpression of SLAMF7 (also known as CS1) was associated with MMSET overexpression in t(4; 14) MM cells [34]. Quantitative RT-PCR and ChIP analysis indicated that MMSET might regulate the transcription level of SLAMF7 and be an important functional element for* SLAMF7* promoter activity.

**Figure 2:**
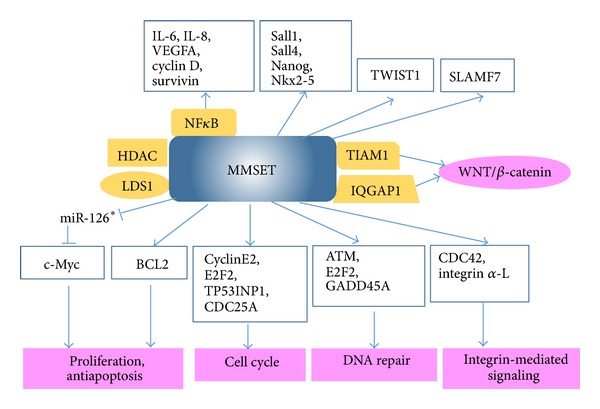
*MMSET interacting proteins and downstream targets.* MMSET interacts with its partners and activates oncogenic signalling pathways, which establish causal roles for MMSET in driving cancer initiation, development, and survival. Orange boxes: MMSET interacting proteins. White boxes: MMSET target genes.

Nimura et al. found that MMSET is associated with the cell-type-specific transcription factors Sall1, Sall4, and Nanog in embryonic stem cells (ESCs) and Nkx2-5 in embryonic hearts [29]. These results suggested that MMSET functions together with developmental transcription factors to prevent the inappropriate transcription that can lead to various pathophysiologies. Immunoprecipitation combined with mass spectrometry analysis revealed IQGAP1 and TIAM1 as candidate interacting partners with MMSET, and these interactions were confirmed by coimmunoprecipitation [35]. IQGAP1 and TIAM1 are both involved in the WNT signaling pathway through interaction with *β*-catenin protein. Gene expression array and real-time PCR analysis indicated that the expression levels of CCND1, an established downstream target of the *β*-catenin/Tcf-4 complex, were reduced significantly upon MMSET knockdown. ChIP analysis showed that MMSET bounds the promoter region of CCND1 [35]. These results suggested that MMSET may regulate the WNT signaling pathway through interaction with *β*-catenin. Yang et al. reported that MMSET is a strong coactivator of NF*κ*B by directly interacting with NF*κ*B for activation of target genes, including those for interleukin-6 (IL-6), IL-8, vascular endothelial growth factor A (VEGFA), cyclin D, Bcl-2, and survivin, in castration-resistant prostate cancer (CRPC) cells [36]. They also found that MMSET is critical for cytokine-induced recruitment of NF*κ*B and acetyltransferase p300 and histone hyperacetylation. Ezponda et al. reported that MMSET could activate TWIST1 to promote an epithelial-mesenchymal transition and invasion in prostate cancer [37]. Whether the aforementioned MMSET interacting proteins and downstream targets in ESCs and solid tumors play critical roles in t(4; 14) MM remains to be determined. The MMSET interacting proteins and downstream targets were summarized in Figure 2.

## 5. Potential Therapeutics Targeting MMSET or Its Downstream Signalling

MMSET overexpression is a universal feature of t(4; 14) MM. Furthermore, t(4; 14) MM cells rely on MMSET expression for proliferation, survival, and tumorigenicity* in vivo*. These findings highlight MMSET as an attractive target for the treatment of t(4; 14) MM. HMT activity of MMSET is essential for its oncogenic function, so it would seem straightforward to design small molecular inhibitors to target MMSET substrate binding pocket. However, very few lead compounds have been published to selectively inhibit MMSET catalytic function. The development of MMSET inhibitors might be hampered by the lack of crystallographic structural information on enzyme-substrate complexes. Recently Cao et al. reported a novel strategy for selective targeting of HMT activity [38]. Instead of directly targeting the catalytic SET domain, they exploit the unique regulation of the MLL1 complex by WDR5 and target MLL1 complex assembly without affecting other MLL family HMTs. As a result, the compound MM-401 shows no inhibition for global H3K4me and little toxicity for normal cells. Transcriptome analyses also confirmed the remarkable selectivity of MM-401, which induces changes in gene expression that are highly correlative with MLL1 gene deletion. MMSET is multidomain protein and several of its partners have been identified, so it is a promising strategy to target MMSET complex assembly for inhibitor development. Numerous HDAC inhibitors are being developed, some of which are at clinical trial stages for MM therapy [39]. These HDAC inhibitors might be promising candidates to inhibit MMSET complex assembly, because MMSET associates with HDACs in a large complex [19, 27, 31].

Sequence-specific gene silencing with small interfering RNA (siRNA) has transformed basic science research, and the efficacy of siRNA therapeutics toward a variety of diseases is now being evaluated in preclinical and clinical trials [40]. The key therapeutic advantage of using siRNA lies in its ability to specifically and potently knock down the expression of disease-causing genes of known sequence. However, clinical use of siRNAs encounters one of the obstacles: delivery of siRNAs to the appropriate cells. Antibody-mediated delivery is an effective method of targeting siRNA to particular cells [41]. Our study showed that SLAMF7 overexpression in t(4; 14) MM was associated with MMSET expression [34]. Thus, it is potential to develop t(4; 14) MM targeted therapy by SLAMF7 antibody mediated MMSET siRNA delivery. This therapeutic strategy will achieve two levels of targeting for t(4; 14) MM: tumor cell selective delivery by the SLAMF7 antibody and gene pathway selectivity by the MMSET siRNA. Furthermore, it was found that, similar to the effects of knocking down MMSET, overexpression of miR-126* could inhibit proliferation of t(4; 14) MM cells* in vitro*. It will be appealing to test whether expression of miR-126* can cause therapeutic effects* in vivo*.

## 6. Conclusion 

The outcome of MM patients has dramatically improved in recent years and this has been possible essentially due to the introduction of the new active agents thalidomide, bortezomib, and lenalidomide, autologous stem-cell transplantation (ASCT), and improvements in supportive care [42]. The median survival is in excess of 5 years. Nevertheless, MM is still considered an incurable disease in the vast majority of patients and the classical pattern of evolution of the disease is of subsequent responses/relapses, with each relapse generally being of shorter duration than the previous ones [43]. Therefore, additional potent therapeutic strategies are urgently needed. The translocation t(4; 14) is one of the most common translocations in MM and is associated with very poor prognosis. MMSET overexpression characterizes all t(4; 14) MM patients, and furthermore MMSET protein is required for t(4; 14) MM cell survival* in vitro* and* in vivo*. The key role of MMSET in t(4; 14) MM allows speculating that a specific pharmacological therapy, which targets this protein, may constitute a novel approach to the treatment of MM.
